# 3D Printing in Solid Dosage Forms and Organ-on-Chip Applications

**DOI:** 10.3390/bios12040186

**Published:** 2022-03-22

**Authors:** Tarek Kassem, Tanoy Sarkar, Trieu Nguyen, Dipongkor Saha, Fakhrul Ahsan

**Affiliations:** Department of Pharmaceutical & Biomedical Sciences, College of Pharmacy, California Northstate University, Elk Grove, CA 95757, USA; tarek.kassem@cnsu.edu (T.K.); tanoy.sarkar@cnsu.edu (T.S.); trieu.nguyen@cnsu.edu (T.N.); dipongkor.saha@cnsu.edu (D.S.)

**Keywords:** 3D printing, organ-on-chip, biosensing, personalized medicine, spritam, CAD, additive manufacturing

## Abstract

3D printing (3DP) can serve not only as an excellent platform for producing solid dosage forms tailored to individualized dosing regimens but can also be used as a tool for creating a suitable 3D model for drug screening, sensing, testing and organ-on-chip applications. Several new technologies have been developed to convert the conventional dosing regimen into personalized medicine for the past decade. With the approval of Spritam, the first pharmaceutical formulation produced by 3DP technology, this technology has caught the attention of pharmaceutical researchers worldwide. Consistent efforts are being made to improvise the process and mitigate other shortcomings such as restricted excipient choice, time constraints, industrial production constraints, and overall cost. The objective of this review is to provide an overview of the 3DP process, its types, types of material used, and the pros and cons of each technique in the application of not only creating solid dosage forms but also producing a 3D model for sensing, testing, and screening of the substances. The application of producing a model for the biosensing and screening of drugs besides the creation of the drug itself, offers a complete loop of application for 3DP in pharmaceutics.

## 1. Introduction

Environmental and genetic variations between individuals result in differences in treatment response to the same targeted agent [1]. Unique means have been developed, such as 3D printing (3DP), to personalize therapies to alleviate the challenges associated with standard medicine dosing. 3DP, described as additive manufacturing, is an emerging individualized oral drug delivery system that researchers are delving to produce pharmaceutical products with individualized doses [2]. Continuous research efforts in this technology resulted in FDA approval of an anti-epileptic drug, spritam (levetiracetam). The significant advantage of this technology is that it provides the room to adjust the dosage according to the unit on demand by altering the object’s geometry or other physical dimensions under observation [3]. The prerequisite of designing any 3D printed dosage form is to develop the substance via Computer-Aided Design software (CAD). CAD provides a pathway to understanding the potential of 3DP for personalized drug therapy of active pharmaceutical ingredients [4]. The CAD programs are used to convert the 3DP file into a stereolithography file (STL file) that possesses the necessary information for the spatial geometry of the object to be printed. After the initialization, the STL file is cut into different segments, one of which is the slice file (SLI segment), which is then uploaded to the 3D printer for printing. The 3D printer acts as a guide for the motion to build the necessary parts. 3DP is one of the novel techniques that allows us to fabricate the oral dosage form with an exact formulation for organ-specific delivery of active pharmaceutical ingredients (APIs) to patients [5]. Although 3DP is in its very early age for being used in the field of personalized drug therapy, it can provide an unprecedented advantage in healthcare settings for designing customized pharmaceutical products and extemporaneous dosage forms [6]. 3DP technology is now expected to revolutionize the industrial setup for both pharmaceutical and non-pharmaceutical products [7]. Over the years, several 3DP techniques have been developed, including stereolithography apparatus (SLA) [8], digital light production (DLP) [9], continuous liquid interface production (CLIP) [10], selective laser sintering (SLS) [11], selective heat sintering (SHS) [12], big area additive manufacturing (BAAG), fused filament fabrication/fused deposition modeling (FFF/FDM) and laminated object manufacturing (LOM), etc. [7]

Existing methodologies for the fabrication of tablets involve a series of steps that include blending and grinding of drugs and pharmaceutical adjuvants, processing, and coating of powders to granules, and finally compression. For the controlled release of drugs, some tablets need further processing, such as making holes within the tablets. Current tablet manufacturing equipment has a fixed set of parameters and thus requires modifications or new tools if tablets require any changes in the dose, size, and shape. Because current instruments require large manufacturing facilities and weighs thousands of pounds, we cannot use these instruments for small-scale fabrication and for preparing dosages to cater to elderly patients with impaired liver or kidney functions or even children, who usually require smaller doses. When reduced doses are required, tablets can potentially be split into pieces and weighed before administration. However, this practice is likely to result in inaccurate dosing. 3DP technology will reduce processing steps for the fabrication of tablets. Using computational design, pharmacists can potentially prepare pills based on patients’ needs. It may sound over ambitious, but it is absolutely possible that pharmacists, one day, will be able to fabricate and dispense tablets in hospitals, war zones, and shelters for war-afflicted populations, areas affected by natural disasters and national emergencies.

This review will provide an overview of different 3DP techniques, 3D printed solid dosage forms, use of 3DP to develop organ-on-chip application, limitations of 3DP techniques, and potential solutions to those limitations.

## 2. 3DP Techniques

There are three major types of 3DP techniques: laser-based printing systems, inkjet-based printing systems, and extrusion-based printing systems [13].

### 2.1. Laser-Based 3D Printing Systems

Laser-based 3DP is primarily of two major types

1. Stereo-Lithography Apparatus (SLA) and
2. Selective Laser Sintering (SLS).

#### 2.1.1. Stereo-Lithography Apparatus (SLA)

**Figure 1:**
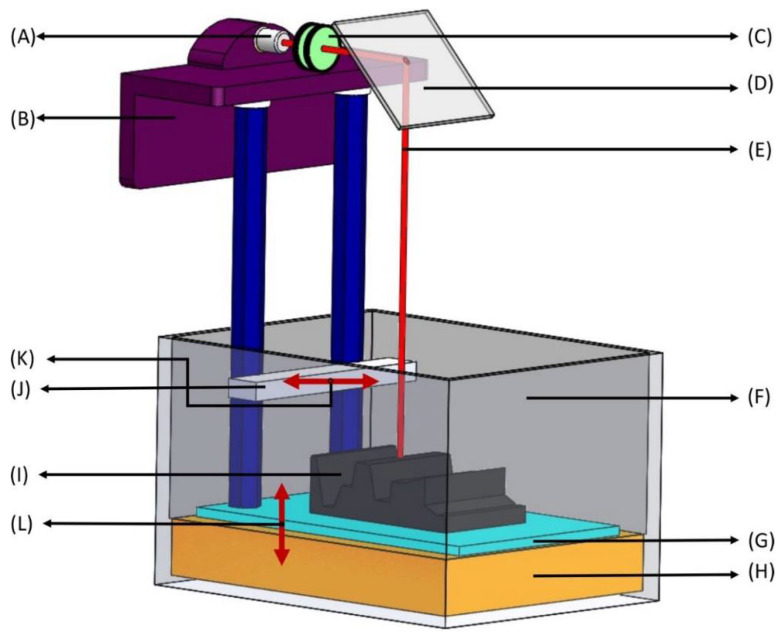
Stereolithography apparatus (SLA). (**A**) Laser—ultraviolet (UV) radiation source with low power; (**B**) Elevator—moves the build platform (shown in **G**) up during production as the laser beam solidifies bottom layers to top layers; (**C**) Lenses—concentrates the UV source to a focused laser beam to treat the surface of liquid photopolymer; (**D**) X-Y scanning mirror—deflects the beam from traveling horizontally to vertically downwards onto the liquid photopolymer (deflection of the mirror depends on the desired 3D-model); (**E**) Laser beam—liquid photosensitive polymers solidify upon contact with the laser beam, leaving untreated polymers liquid; (**F**) Vat—a large container holding photosensitive liquid; (**G**) Build platform—layers of photosensitive polymers are produced layer-by-layer from bottom to top and each layer represents a cross section of the final 3D product at every 50–200 μm; (**H**) Liquid photopolymer—photopolymers consist of a mixture of multifunctional monomers and oligomers; (**I**) Layered part—a combination of solidified photopolymer layers; (**J**) Sweeper—recoats the surface of the layered part with a liquid photopolymer resin as the build platform (in **G**) moves down after the solidification of each layer; (**K**,**L**) Motion of parts—right and left movement of the sweeper (in **K**) and up and down movement of the build platform (in **L**).

SLA was one of the first 3DP technologies to be invented by Hull in 1986 in which radiation is applied on some photo-sensitive polymers to initiate the process of photopolymerization [14]. Digitally controlled UV-Light emitters are usually utilized to scan the surface of liquid polymers and plastic resins, which are photo-polymerizable. After polymerization, the 3D printer creates a layer of solid resins equivalent to the depth of the previous layer of polymer [15]. The excellent penetration potential of UV light causes the fusion of multiple layers of polymers. These cycles are repeated several times to achieve the intended design of a dosage form [13]. Figure 1 shows the design of the stereo-lithographic apparatus. Continuous liquid interface production (CLIP) is a modified version of SLA. Unlike SLA, the polymerization process in this technique is ongoing and continuous instead of a layer-by-layer pattern, and it requires a pool of liquid photopolymer resin [16]. In CLIP, the printing speed and resolution are very high compared to traditional SLA [17] and can create objects nearly 100 times faster than the other 3DP methods commercially available [16]. This method has a limited application in the pharmaceutical business due to the increased energy input from the laser [18,19].

#### 2.1.2. Selective Laser Sintering (SLS)

**Figure 2:**
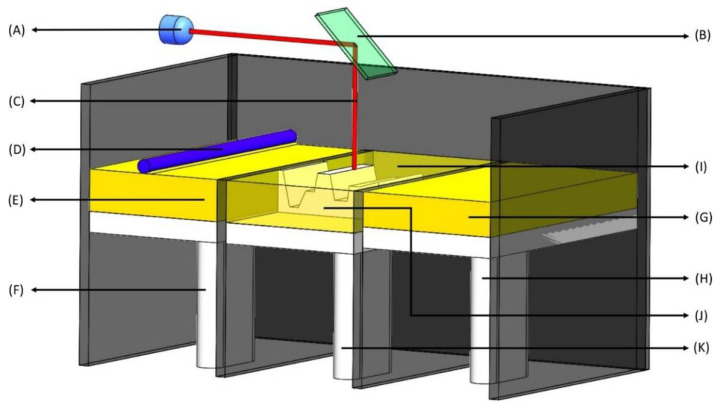
Selective laser sintering (SLS). (**A**) Laser source—UV radiation source; (**B**) X-Y scanner—changes X and Y coordinates for laser treatment based on the 3D model; (**C**) Laser—high power laser to sinter small polymer particle into solid structure; (**D**) Roller—produces single layers of powder; (**E**) Raw material—a powdered form of raw material such as polymers; (**F**) Recoater piston—travels upwards in one layer succession providing a new layer of powdered material; (**G**) Powder delivery system—a reservoir of powder that is delivered to the build chamber (in **I**); (**H**) Powder delivery piston—is raised to deliver the new layer of material; (**I**) Build chamber—powder is dispersed in a thin layer on top of the platform inside the build chamber; (**J**) Layered part—the final 3D product which is combination of all the layers; (**K**) Build piston—is lowered to add a new layer of material.

SLS is one of the emerging technologies in the field of 3D Printing. It involves only a single step in which a laser selectively sinters powders into layers to achieve the intended 3D structures. This technique consists of using focused lasers on the surface of powders to draw specific patterns by stacking powder materials. As the layers are being sintered, the powder beds move downward, and the reservoir beds move upward to make new layers, and the new layers are then stacked up on the previous layers (Figure 2). The process is repeated several times to achieve the intended dosage designs. Different types of polymers have been employed to produce pharmaceuticals, such as thermoplastic materials like PA12 (Nylon) and polyether ether ketone (PEEK) [20]. The SLS process has been used to make orally disintegrating tablets such as ondansetron [21].

### 2.2. Inkjet-Based 3D Printing Systems

**Figure 3:**
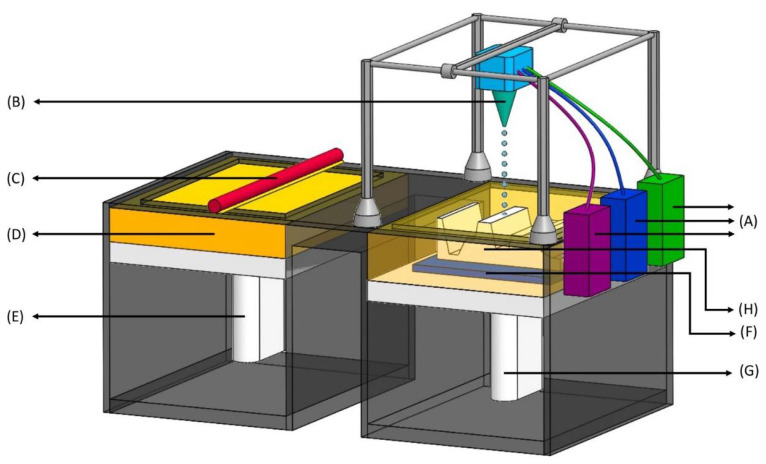
Inkjet printing process—drop-on-powder (DoP). (**A**) Binder feeders—reservoir of liquid ink containing polymers; (**B**) Inkjet printhead—contains a series of nozzles that dispenses a continuous stream of ink, printing in patterns to produce structures, and unused ink is recirculated back to the reservoir (in **A**); (**C**) Leveling roller—pushes a layer of raw material onto a powder bed; (**D**) Powder supply—reservoir of powder; (**E**) Powder feed piston—pushes up new layers of powder to leveling roller as the build piston (in **G**) comes down; (**F**) Powder bed—where the layered part (in **H**) is built; (**G**) Build piston—comes down after each subsequent layer is formed on the powder bed; (**H**) Layered part—the final product which is a combination of all the layers.

**Figure 4:**
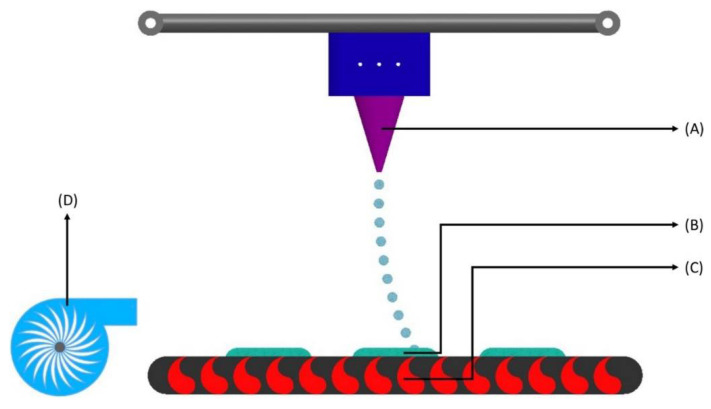
Inkjet printing process—drop-on-drop (DoD). (**A**) Optical adjuster—provides discontinuous droplets through short pressure wave pulses; (**B**) Layered part—the final product which is a combination of all the droplet-based layers; (**C**) Heat source—heat generates small air bubbles that activate the release of droplets; (**D**) Blower—directs heated air flow towards the layers of the ink composition.

Inkjet-based Printing is one of the most-used 3DP techniques, which is further subdivided into two major types: Drop-on-Powder (DoP) deposition and Drop-on-Drop (DoD). DoP deposition uses either a powder bed covered with unbound powder material or inkjet printing to jet a liquid binder on to a powder bed to generate 3D structures (Figure 3). On the other hand, in DoD, the liquid droplets are the building materials placed on the surface of a substrate in a coherent pattern (Figure 4). The API can be dissolved in a liquid medium that acts as a binder or is formulated into powders that serve as the powder bed. The principle of interaction between the binding liquid and the powder bed is like the wet granulation technique [22]. The Drop-on-Powder technique is more suitable to produce pharmaceuticals [23].

### 2.3. Extrusion-Based 3D Printing Systems

**Figure 5:**
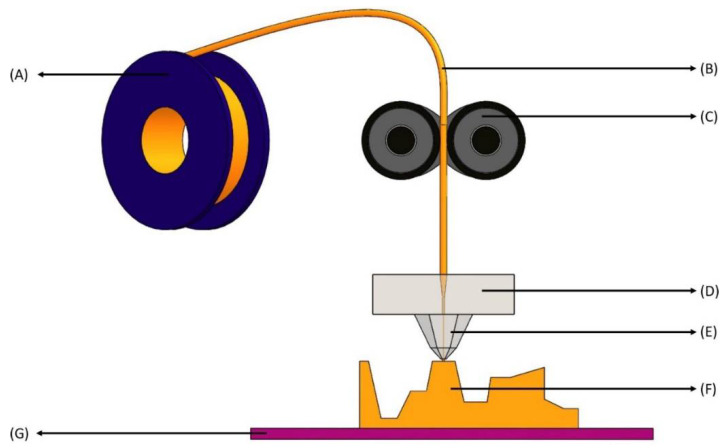
Fused filament fabrication printing process. (**A**) Filament spool—holds the filament and can be easily replaced with new spools; (**B**) Filament—comes from spool to the roller extruder (shown in **C**) and is the principal supply of material in this technique; (**C**) Roller extruder—the extruder pinches the filament as it enters and uses torque to feed or retract the precise amount of filament; (**D**) Heat extrusion head—contains a heater block that melts the filament to a desirable temperature for use; (**E**) Nozzle—squeezes the heated filament with smaller diameter; (**F**) Layered part—as the nozzle places the extruded material, the final product forms layer by layer; (**G**) Print bed—moves as the final product is being formed depending on the solid 3D model.

Extrusion-based printing systems are also known as nozzle-based printing systems, an ‘additive’ technology used in modeling, prototyping, and production applications. Its printing process is quite similar to that of laser-based and inkjet-based systems. It requires a plastic filament as the primary printing material. It lays down plastic material layer-by-layer to form a 3D object utilizing a bottom-up construction approach. These systems are classified based on the need for the heating system to melt the powder or not and can be of two major types: (i) pressure-assisted micro-syringes (PAM) and (ii) fused filament fabrication (FFF) (Figure 5) [13].

#### 2.3.1. PAM

PAM is the technique in which the powder and binder are mixed to make a semi-solid material extruded at a pressure of around 3–5 bars. The material is not immediately solidified; rather, it requires exposure to light or air to completely harden the material [24]. This is one of the reasons why there are risks in shrinking or deformation of the intended structure, or if the material is not hardened enough, then there are chances of collapsing the whole structure [18]. In PAM, certain solvents are utilized to produce a semi-solid material. After evaporation at room temperature, the solvents generate the intended final product. These solvents are often toxic and sometimes may create unnecessary damage to API by altering its stability profile [15].

#### 2.3.2. FFF

Many authors also recognize FFF as fused deposition modeling (FDM), Table 1. This technique has gained widespread acknowledgment in both pharmaceutical and non-pharmaceutical fields. It is mainly employed in the pharmaceutical field to produce an oral dosage form by thin layer deposition of the material [25]. A continuous filament of thermoplastic material is utilized as a solid filament and is fed to a moving and heated printer extruder head via a gear system. The material is converted into a soft substance in the print head before being extruded via a nozzle system. After extrusion, the extruded material solidifies virtually quickly. FFF/FDM 3DP technology, although expensive, is user-friendly and extremely simple [18].

## 3. Polymers Used in 3D Printing of Pharmaceutical Solid Dosage Form

3DP utilizes different polymers and polymer combinations to produce novel solid dosage forms. Polyvinyl alcohol (PVA) and polyvinylpyrrolidone (PVP) are the most widely used polymers. Polymers used in 3DP are usually classified as non-biodegradable such as PVA, polyethylene glycol (PEG), Eudragit L 100, etc.; biodegradable such as poly L-lactic acid (PLLA), polycaprolactone (PCL), etc.; and amalgams. Polymer amalgams combine two or more polymers such as Eudragit RL PO plus PLA [26]. A list of polymers and their combinations used in 3DP are listed in Table 2.

## 4. 3DP Solid Dosage Forms

Researchers have been employing 3DP to produce pharmaceutical solid dosage forms and others for several decades. However, currently, there is only one formulation of levetiracetam (spritam) available on the market. FDA approved spritam in 2015. The drug is formulated as fast disintegrating tablets available in 4 different strengths, i.e., 250 mg, 500 mg, 750 mg, and 1000 mg. In 2020, Giomouxouzis et al. published a study and used the FDM 3DP method in which they utilized diltiazem as the model drug for preparation of diltiazem caplets using PVA and cellulose acetate (CA) as the polymers for ink. Thermal analysis techniques (TGA, DSC) are used to assess the physicochemical properties of the prepared caplets and X-ray diffraction (XRD) and scanning electron microscopy for analyzing the morphological features. The XRD analysis shows amorphization of diltiazem inside the polymer [49]. In 2019, Gültekin et al. utilized FDM as the 3DP method and Eudragit EPO + POLYOXTM WSR N10 and Eudragit EPO + POLYOXTM N80 as polymers to prepare tablets and filaments of pramipexole dihydrochloride monohydrate. Scanning electron microscope (SEM), differential scanning calorimetry (DSC), and filament disintegration tests were used to assess the characteristics of prepared filaments and tablets [50]. In 2020, ondansetron and anti-emetic drugs were used as model drugs to prepare orodispersible printlets in which Kollidon VA-64 was used as the major polymer, and selective laser sintering (SLS) was the method of choice for 3DP [21].

Many drugs have been investigated to convert them into a novel solid dosage form by utilizing 3DP technology. Khaled et al. used a pressurized micro syringe technique (PAM) as the printing technology to develop a novel solid dosage form involving three different drugs—captopril, nifedipine, and glipizide. Each of the three medications has a unique release profile. Captopril and nifedipine are well-known antihypertensive drugs, while glipizide is typically used to treat type 2 diabetes [24]. Goole and Amighi in 2016 [15] further investigated the study mentioned above. Hydroxypropyl cellulose (HPMC) was used as a primary polymer for glipizide and nifedipine. Both drugs were dispersed in HPMC but in separate compartments. On the other hand, for captopril, the polymers used were PEG 6000 and cellulose acetate (CA). These polymers are used to create a porous system through which the drug is released via osmotic diffusion. The only drawback of this technique was an increased tablet size, which creates difficulties in swallowing [15].

Recently, Tabriz et al., 2021 developed a novel solid dosage form (tablets) for isoniazid and rifampicin. Both isoniazid and rifampicin are used to treat tuberculosis as the first line of therapy. HPMC is the polymer of choice for isoniazid, whereas hydroxymethyl propyl cellulose acetate succinate (HMPCAS) is the polymer of choice for rifampicin. Both drugs are printed in two different layers, and then those two layers are fused to make a single tablet [51].

Ibrahim M et al., 2019 used metformin HCl as a model drug to prepare metformin tablets using FDM as 3DP technology in which PVA filaments are used as a polymer of choice. To enhance the solubility of the drug, ethanol is utilized as a solvent to prepare the solution of metformin HCl (low water content, i.e., 10% *v*/*v*). Afterward, the PVA filaments are soaked in metformin HCl/ethanol solution for a specified time. Then, the solutions are aliquoted in several vials and continuously stirred for 1, 3, 6, and 10 days to achieve the maximum drug loading. SEM, X-ray powder diffraction (XRPD), Fourier Transform Infrared Spectroscopy (FTIR), DSC, and dissolution studies are used to physiochemically characterize the prepared metformin-PVA (ML-PVA) filaments [52]. Similarly, in another study by Saviano M et al., 2019, FDM 3DP is used to prepare ciprofloxacin HCl + PVA’s drug-loaded filaments. The dried powder of both the drug and the polymer is mixed to prepare the physical blends. Dibutyl sebacate is added along with drug and polymer to increase drug adhesion on the pellets and facilitate the extrusion process. The drug-loaded filaments of diameter 2.85 ± 0.15 mm are used to feed the extruder, resulting in flat-faced cylindrical printlets [53]. Table 3 includes a list of drugs where 3DP technology is utilized to convert into novel solid dosage forms.

## 5. 3D Printing for Organ-on-Chip Application and Drug Sensing

After producing oral dosage forms, it is essential to test the drugs in a model, such as conventionally used animal models. However, the animal model often does not recapitulate the entire physiology of a human body. It is also challenging to study cell–cell interactions with animal models. There are also increased ethical concerns about using an animal model in society [66].

With the current advancement in microfabrication such as photolithography and 3D printing, scientists have engineered 3D models called organ-on-chip (OoC) systems, which give the capacity of not only mimicking the cellular/tissue level in its microenvironment but also of acting as a systematically analytical tool for disease progression. For a definition, OoC is the technology that aims to create artificial living organs, which are then used to mimic the physiological responses of the actual organs. This technology offers a realm for drug testing, sensing and accurately manipulating cells in an in-vivo-like manner.

### 5.1. 3D Printing for Microfluidics

Microfluidics refers to the technology of liquid handling in tiny channels with dimensions in the order of one to ten micrometers [67,68]. This field has emerged in the last 30 years due to its application in several diverse fields, namely medicine, biology, chemistry and physics. The microfabrication techniques used to create these microchannels mainly originated from microelectronic areas, i.e., silicon technology. At first, the microfluidic chips can be made from silicon wafers, then glass [69] and fused silica [67] using dry and wet etchings, and currently polymers [70,71] using polymer injection molding and hot embossing.

3D printing offers a new platform to create microstructures and channels with dimensions in the order of 10 microns. It is an innovative add-on fabrication technique that grants the engineering of lab-on-a-chip devices that are occasionally difficult to pattern using traditional approaches such as micromachining or molding [72].

### 5.2. 3D Printing for Tissues and Organs

3D bioprinting is a fabrication process in which tissues are printed three-dimensionally. The ink used for bioprinting is usually called bio-ink. The bio-ink comprises living cells when printing tissues or organs, and in the case of printing scaffolds, it contains biomaterials such as agarose, alginate, collagen, cellulose and so on [73].

3D printing has been a promising candidate for the fabrication of an OoC platform due to its precise control of layer-by-layer assembly of biomaterials, such as an extracellular matrix (ECM), cells, etc. [74] While it is still in the early stages, 3D cell printing has displayed promising utilities in screening, testing drugs by modeling tissues and diseases [75], including skins [76], cancers [77], liver [78], lung [79], etc.

For example, Nguyen and co-authors reported [78] bio-printed 3D human liver tissues possessed of primary human parenchymal (hepatocyte) and non-parenchymal (endothelial and hepatic stellate) cell populations, which were then assessed for their possible use as substantial, multi-cellular models of human liver tissue. The authors showed the primary histologic, biochemical, and metabolic properties of the 3D liver tissues. Furthermore, to investigate the ability of the tissues to be used as a model of drug-induced liver injury, the authors tested their model response to the known hepatotoxicant Trovafloxicin in comparison to its harmless corresponding Levofloxacin. In general, the results emphasized that the 3D liver tissues formed by the 3D bioprinter can be a beneficial add-on to the pre-clinical toxicity studies.

Another example, presently, Kang and co-authors, has succeeded in producing an artificial lung model using 3D printing [79]. They claimed that this 3D alveolar barrier model can be used as a replacement for conventional models for pathological and pharmaceutical applications.

### 5.3. 3D Printing for a Complete Organ-on-Chip

3D printing usually serves as a tool to print either microfluidic devices or tissues, as shown in the previous sections. An organ-on-chip device should have both microfluidic channels which function as a microenvironment, and engineered tissues [80] to mimic the physiology of human organs. It has recently become possible to print both channels and cells using 3D printers. For example, in a single-step fabrication, all factors of the 3D tumor models such as the microfluidic channels, body of the chip, and the 3D tumor tissues are produced straight from the inputs of a user. This can be manifested by using multiple printing heads, allowing both 3D printing and 3D bio-printing [81].

**Figure 6:**
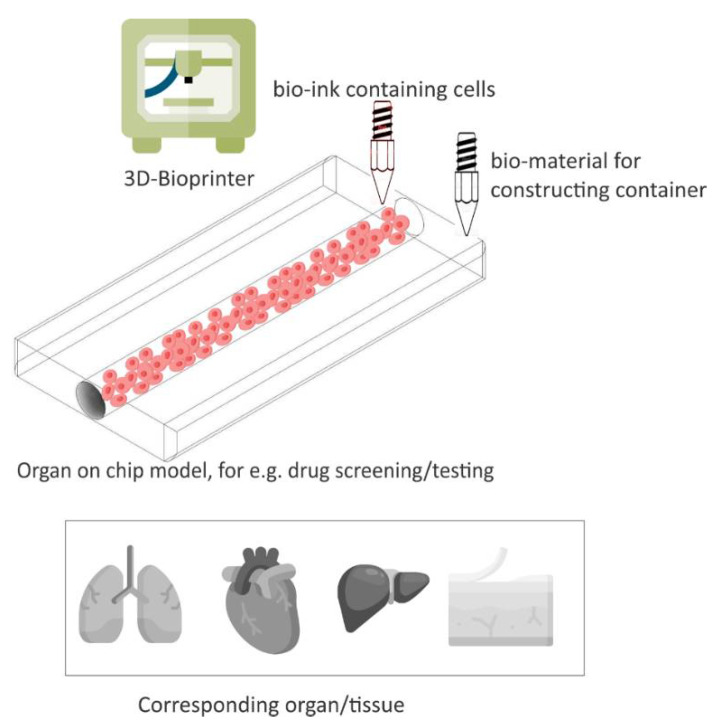
An illustration of 3D bioprinting, which provides 3D cell culture OoC devices for drug screening/testing. Adapted from [74].

Figure 6 presents an illustration of 3D bio-printing for organ-on-chip applications, e.g., drug screening/testing. 3D printing technology has hence not only offered the tools for producing oral dosage forms, but has also been shown to be capable of providing platforms such as OoC for drug screening, sensing, and testing, making it a perfect candidate for pharmaceutical applications.

### 5.4. Pharmaceutical Application

Drug development is time-consuming and expensive via clinical trials [82]. The essential goal of the OoC field is to boost and enable assessment in drug discovery and development [83]. This ultimate desire has fueled the establishment of many start-ups and spin-off companies that have conveyed the research realm mainly in academia towards efficient and reliable commercially availability on a product or service basis.

One of the examples in the pharmaceutical productions of the 3D bioprint OoC is the use in testing the toxicity of pre-clinical drug candidates as doing so by Organovo Inc., in San Diego, CA, USA. Organovo Inc. has put up a robust business as a contract research organization that evaluates experimental drug compounds on the 3D-printed liver. Top global pharmaceutical companies, such as Merck, Bristol-Myers Squibb, and Roche are now using the service of Organovo Inc.

## 6. Limitations of 3DP Technology

3DP technology is no doubt a revolutionary technology in the field of both pharmaceutical and non-pharmaceutical industries. With each passing year, breakthroughs are being made, such as techniques being improved to solve flaws and new materials being tested to overcome material limitations for the cost-effective production of pharmaceuticals using any 3DP methods. At present, 3DP technology is experimental. Still, it has one significant advantage over conventional processes, i.e., with this technology, it is possible to bring the production of personalized medicines closer to patients in local small-scale pharmacies and hospital settings. However, every technology has its own merits and demerits. One of the significant demerits of 3DP is that it takes a lot of time to produce only a modest amount of product. For instance, the tableting process produces ~15,000 tablets per minute using a single press machine with conventional techniques. On the other hand, 3D printing of tablets is time-consuming. Usually, the production time for a single tablet varies from 2 min to 2 h [15]. Due to this limitation, there are very few industrial applications of this technology since producing a large number of products is time-consuming. Moreover, the process is energy-dependent, i.e., the time taken for each batch production is directly related to energy consumption. Due to these challenges, industrial applications are minimal at present. Consistent efforts are being made to overcome the barriers that hinder the applications of 3DP in the pharmaceutical industry.

## 7. Conclusions

For several years now, 3DP technology has gained significant attention from researchers. Researchers are now starting to understand the potential of this technology for the production of individualized novel solid dosage forms. Spritam (Levetiracetam), one of the 3D printed solid dosage forms, was authorized by the FDA in 2015. Out of the many 3D printed methods, FDM has proven to be more significant to produce pharmaceuticals and has been vastly researched. This technology can revolutionize the pharmaceutical industry by creating a doorway for the possibility of achieving personalized medicines. As this technology has many advantages, it also possesses significant demerits that cannot be overlooked. Restricted material usage, slow production process, and unreacted material in the final product are some of the significant drawbacks of this technology. In the future, consistent efforts are needed to alleviate these shortcomings. Overall, the potential of this technology in the healthcare sector is undeniable as it can help us achieve the seemingly difficult task of personalized medicine. It will be interesting to see how much time and work it takes to make it available to all healthcare settings and to the pharmaceutical sector.

## Figures and Tables

**Table 1:** A summary of different types of 3DP technologies.

3DP Methods	Types	3DP Process	Polymers Used	Disadvantages
Laser-based printing system	Stereo-lithography apparatus	Digitally controlled UV-light emitters are usually utilized to collect the polymers. These UV light emitters scan the surface of the liquid polymers and plastic resins, which are photo-polymerizable. After polymerization, the 3D printer creates a layer of solid resins equivalent to the depth of the previous polymer layer.	- Polyethylene glycol diacrylate (PEGDA) - Poly-2- hydroxyethyl methacrylate - Polyethylene glycol dimethacrylate - Polypropylene fumarate-diethyl fumarate	- Polymers are not generally recognized as safe (GRAS) listed. - High and unselective reactivity of the polymers. - Poor solubility of API in polymer solution causes sedimentation issues
	Selective laser sintering	This technique involves using focused lasers on the surface of powders to draw specific patterns by stacking powder materials. As the layers are being sintered, the powder beds move downward, and the reservoir beds move upward to make new layers, and the new layers are then stacked up on the previous layer.	- Polyether ether ketone (PEEK) - PA12 (Nylon)	- Material is restricted to laser absorption by the components. - Hollow object printing is not possible - Decomposition of components occurs due to high energy input of laser
Inkjet-based printing systems	Drop on Demand printing	A Drop-on-Powder deposition is an inkjet-based technique that uses either a powder bed covered with unbound powder material or a mechanism consisting of a powder jet. The API can be dissolved in a liquid medium that would act as a binder or formulated into powders that serve as the powder bed. The principle of interaction between the binding liquid and the powder bed is similar to the wet granulation technique.	- Microcrystalline cellulose (MCC) - Spray-dried lactose - Maltitol - Maltodextrin - Polyvinyl pyrrolidone	- Organic solvents are used, which may be toxic - Additional drying is required - Resolution is dependent on the particle size of the polymer - Hollow object printing is not possible
Extrusion-based printing systems	PAM	PAM is the technique in which the powder and binder are mixed to make a semi-solid material extruded at a pressure of around 3–5 bars. The material is not immediately solidified. Instead, it requires exposure to light or air to harden completely.	- Polycaprolactone (PCL) - Poly lactic-co-glycolic acid (PLGA) - Poly-L-lactic acid (PLLA)	- Drying step is required - Frequent utilization of organic solvent - The nozzle diameter limits resolution
	FFF/FDM	Thermoplastic starting materials are utilized as solid filaments, fed to the print head via a gear system. The material is converted into soft material in the print head before being extruded via a nozzle system. The extruded material solidifies almost instantly after extrusion.	- EC+ Eudragit^®^ L100 - HPMC + polylactide acid (PLA) - Polyethylene oxide (PEO) - Thermoplastic polyurethane	- Materials restricted to thermo- plastic polymers - Manufacturing of raw material needed - High process temperatures - Application restricted to thermostable APIs - Printing resolution limited by nozzle size

**Table 2:** Non-biodegradable polymers and their combinations employed for the production of pharmaceuticals via 3DP technologies.

Name of Polymer	3DP Technology	Employed Hot Melt Extruder	Dosage Form	Refs.
**Polyvinyl alcohol (PVA)**	FDM/FFF	Single screw extruder (SSE)	Tablets	[27,28,29,30]
		Conical screw extruder (CE)	N/A	[31]
**Polyvinyl pyrrolidone-vinyl acetate copolymer (Kollidon VA-64)**	FDM/FFF	SSE	Tablets	[32]
		Co-rotating twin screw extruder (TSE)	Tablets	[33,34]
		Conical screw extruder (CE)	N/A	[31]
		Ram extruder (RAM)	Tablets	[35]
**Polyvinyl alcohol-polyethylene glycol graft copolymer (Kollicoat IR)**	FDM/FFF	SSE	Capsules	[36]
		RAM	Tablets	[35]
		CE	Discs and capsule shell	[37,38]
		TSE	Tablets	[34]
**Poloxamer-407**	FDM/FFF	RAM	Tablets	[35]
**Polyether ether ketone (PEEK)**	SLS	N/A	Tablets	[20]
**PA12 (Nylon)**	SLS	N/A	Tablets	[20]
**Polyethylene glycol (PEG)**	FDM/FFF	RAM	Tablets	[35]
		TSE	Tablets	[35]
**Polyvinyl caprolactam-polyvinyl acetate-polyethylene glycol graft co-polymer**	FDM/FFF	TSE	Discs	[39]
		CE	Tablets	[31,37,40]
**Eudragit E**	FDM/FFF	CE	Tablets	[41]
**Polyethylene glycol diacrylate (PEGDA)**	SLA	N/A	Tablets	[42]
**Poly(2-hydroxyethyl methacrylate)**	SLA	N/A	N/A	[42]
**Polyethylene glycol dimethacrylate**	SLA	N/A	N/A	[43]
**Polypropylene fumarate-diethyl fumarate**	SLA	N/A	N/A	[15]
**Eudragit RL**	FDM/FFF	CE	Capsule shells, tablets	[40]
		SSE	Tablets	[44]
**Eudragit EPO**	FDM/FFF	CE	Tablets, Capsule shells, Discs	[19,40,45]
**Eudragit RL PO**	FDM/FFF	CE	Solid Discs	[37]
		SSE	Oral Solid Dosage form	[26]
		TSE	Tablets	[46]
**Eudragit L 100**	FDM/FFF	TSE	Tablets	[39]
**Eudragit L 100–55**	FDM/FFF	CE	Discs	[37]
		RAM	Tablets	[35]
**Eudragit RS**	FDM/FFF	CE	Tablets	[41]
**Eudragit RS PO**	FDM/FFF	RAM	Implants	[47]
**Microcrystalline cellulose (MCC)**	Drop-on-Powder	N/A	Tablets	[48]
**Spray-dried lactose**	Drop-on-Powder	N/A	Tablets	
**Maltitol**	Drop-on-Powder	N/A	Tablets	
**Maltodextrin**	Drop-on-Powder	N/A	Tablets	

**Table 3:** A list of drugs has been investigated for their conversion into novel solid dosage forms using the 3DP technology.

Name of Polymer	Type of 3DP Technology	Dosage Form	Drug Used	Physicochemical Characterization Methods	Refs.
**Polyvinyl alcohol (PVA)**	FDM	Caplets	Diltiazem	TGA DSC XRD SEM Micro Computed Tomography	[49]
**Cellulose acetate (CA)**	FDM-hot melt extrusion	Caplets	Diltiazem		
**Eudragit EPO + POLYOX™ WSR N10**	FDM-hot melt extrusion	Filaments and tablets	Pramipexole dihydrochloride monohydrate	SEM DSC Filament Disintegration test	[50]
**Eudragit EPO + POLYOX™ WSR N80**					
**Kollidon VA-64**	SLS	Orodispersible printlets	Ondansetron	DSC SEM Micro-CT XRD HPLC	[21]
**Hydroxypropyl cellulose + Vinyl pyrrolidone-vinyl acetate (copolymer)**	FDM-hot melt extrusion	Tablets	Anhydrous caffeine	XRPD DSC Confocal Raman Microscopy	[54]
**PVA**	FDM-hot melt extrusion	Tablets	Ciprofloxacin HCL	SEM DSC	[53]
**Carrageenan + Xanthan gum**	Extrusion based 3D printer	Gummies (Solid dosage form)	Ranitidine HCL	DSC XRD	[55]
**HPMC + K4M**	FDM	Tablets	Theophylline	SEM Textural Profile Analysis (TPA)	[56]
**Hydroxypropyl cellulose (HPC)**	FDM-hot melt extrusion	Tablets	Isoniazid	SEM DSC XRPD Energy dispersive X-ray (EDX) HPLC	[51]
**Hydroxymethyl propyl cellulose acetate succinate (HMPCAS)**	FDM-hot melt extrusion	Tablets	Rifampicin		
**PVA**	FDM	Tablets	Metformin	SEM DSC IR Analysis XRD HPLC	[52]
**Poly(Lactic-co-glycolic acid) PLGA**	Extrusion based 3D printing	Oral solid dosage form (hydrogel discs)	Paclitaxel + Rapamycin	N/A	[57]
			Lidocaine		
**PEG + CA**	PAM	Tablets	Captopril	SEM DSC XRPD	[15,24]
**HPMC**			Nifedipine		
			Glipizide		
**HME + Polymethacrylate-based copolymer** **Or** **HPC + triacetin**	FFF	Tablets	Theophylline	SEM DSC XRPD	[41]
**PLGA + polycaprolactone**	FFF	Bio-degradable implants	5-Flourouracil	SEM DSC XRPD	[58]
**2-Pyrrolidone**	Inkjet-based DoP	Tablets	5-Flourouracil	SEM DSC XRPD	[59]
**PVA**	FDM	Tablets	4-amino salicylic acid	SEM DSC XRPD	[60]
			5-amino aalicylic acid		
**Polyvinyl pyrrolidone**	Extrusion based	Tablets	Paracetamol	XRPD ATR-FTIR DSC	[61]
**Ethyl cellulose**	FDM	Tablets	Ibuprofen	SEM DSC XRPD	[62]
**HPMC + Polyacrylic acid (PAA)**	Extrusion based	Bi-layer tablets	Guaifenesin	SEM DSC XRPD	[24]
**PVA**	FDM	Orodispersible film	Aripiprazole	XRD DSC SEM	[63]
**PVA**	FDM	Tablets	Prednisolone	DSC XRPD SEM	[64]
**Polyvinyl pyrrolidone**	Water-based inkjet	Tablets	Thiamine (Vitamin B1)	SEM DSC XRPD	[65]

## Data Availability

Not applicable.
